# Supplementary material for: Are carbon emissions trading and green financial instruments synergistic? -Comprehensive quantitative research based on content analysis
Source: PLoS One. 2024 Mar 7;19(3):e0298601. doi: 10.1371/journal.pone.0298601 (PMC10919601; doi:10.1371/journal.pone.0298601)
Supplement: S1 Data — (ZIP) [file pone.0298601.s001.zip › Data/地级市Z_F工作报告.zip!/┴╔─■/╕o╦│╩╨/└┤╫╘╣1⁄2╓┌║┼í╛┬φ┐╦╩2╛▌═°í┐_2017.docx]

各位代表:

现在，我代表市政府，向大会报告政府工作，请予审议，并请市政协委员提出意见。

一、2016年工作回顾

过去的一年，是抚顺积极应对严峻复杂形势，保持战略定力、只争朝夕，攻坚克难、奋力前行的一年;是抚顺抢抓新一轮东北振兴重大机遇，贯彻落实新发展理念和“四个着力”要求，积极推进供给侧结构性改革,大力实施“一极五业”发展战略取得扎实成效的一年;是抚顺积极构建科学精细的经济社会管理模式，努力创造宜居乐业的经济社会发展环境，老工业基地转型振兴取得积极进展的一年。

一年来，在省委、省政府和市委的正确领导下，在市人大和市政协的监督支持下，在全市人民的共同努力下，抚顺经济社会平稳健康发展。预计完成地区生产总值1050亿元,全社会固定资产投资155.1亿元，一般公共预算收入77.3亿元，城镇新增就业2.8万人，城镇居民人均可支配收入28587元，农村居民人均可支配收入12530元。全年民生支出占财政支出比重达八成以上，占比居全省前列。

（一）抢抓重大历史机遇，振兴成效不断显现

积极争取政策资金支持。市级领导带头，主动对接新一轮东北振兴战略，进京50余次争取国家部委和央企支持，《抚顺资源枯竭型城市可持续发展示范市总体方案（2015-2020年）》经省政府批准,正式报国家发改委；地质灾害区居民（6141户）避险搬迁安置，争取到国家采沉棚户区改造补助最高标准；西露天矿地质灾害综合治理项目列入科技部2017年重点项目指南；国家测绘地理信息局将我市列为全国首个区域性地理国情监测中心。以省委、省政府大力推进沈抚新区建设为契机，做好了接受政策辐射和配套产业发展准备。中石油集团公司将抚顺石化产业园区列为东北三省重点支持的5家产业园区之首。借国家建设沈佳高铁之机，沈吉铁路（城区段）外迁工作取得突破性进展。全年争取各类上级资金实际到位28.9亿元。

一批重大项目签约落地建设。投资109亿元的清原抽水蓄能电站项目提前一年开工建设。投资34.9亿元的抚顺矿业集团热电机组“上大压小”项目并网发电。投资12亿元的金管家养老服务中心、投资11亿元的丰远热高乐园梦幻世界、投资6亿元的沥青基碳纤维等一批大项目正在建设中。26个亿元以上项目近期有望落地和开工。

全力策划包装项目。围绕老工业基地转型振兴，全口径策划包装项目268个，总投资超过2000亿元。经过不懈努力，全市重大项目储备增多，在招商引资和向上争取中作用凸显。

加大对外开放力度。加强载体建设，全市开发区（产业园区）由26个整合为11个；绘制完成各县区存量土地详图、全市闲置厂房地理位置分布图，盘活利用闲置土地300公顷、闲置厂房10.2万平方米。我市开发区整合和闲置资源盘活工作走在全省前列。先后赴韩国、俄罗斯和珠三角、长三角等国内外地区招商32次，请进来开展招商活动200余次、对接客商2500余人次，与中国核工业建设集团、省水资源管理集团等签订战略合作协议，新加坡、荷兰等国家20余个高端团组来抚经贸交流。全年新签约亿元以上项目合同或协议41个、总投资129亿元。主动融入“一带一路”发展战略，对巴基斯坦、新加坡、越南出口额分别增长3.5倍、1.4倍和1.2倍。借助中国对外承包工程商会、中国联合工程公司等平台，帮助企业开拓国际市场成效显著，抚挖重工出口液压履带式起重机50台、总价2000万美元。

（二）着力完善体制机制，发展活力不断增强

全面深化行政审批制度改革。取消下放行政审批事项46项，行政审批中介服务事项由88项减至67项，建设工程项目审批时限由275个工作日减少到40个工作日，其他审批事项平均办理时限缩短50%。

改进和优化公共服务。成立市行政审批服务局，强力推进“两集中、两到位”，具备行政审批职能的30个市直部门、7个事业单位和4个中省直部门全部进驻，288项行政审批职权全部进厅集中办理。持续开展“换位体验”活动，全市46个机关事业单位改进和优化公共服务事项367项，以“晒清单”形式向社会公开。

强化协调服务职能。分别在市发改委、经信委、科技局设立面向中省直企业、民营企业（中小微企业）、科研院所和大专院校的协调服务中心，协调解决问题400余个。召开全市工业企业协作配套会议，达成大企业与中小企业产品配套协议额16亿元。落实各项减税降费政策，为企业减免退税17.7亿元、减少电费支出5331万元。

加快国资国企改革步伐。完成市直机关所属企业脱钩移交。完成市文化旅游集团和交通产业集团组建工作，完成市城建集团、园林绿化建设集团、国有资本投资运营集团和财金投资控股集团组建方案制定和论证。

稳步推进事业单位改革。推进部分从事生产经营活动事业单位剥离职能、转为企业、组建企业集团，撤并整合职能弱化事业单位，全年减少财政供养人员170人，节省财政支出1751万元。市县两级《国有林场改革实施方案》获省批复。

其他领域改革取得积极进展。推进“营改增”财税体制改革，为企业减税让利基础上，实现地方税收61.2亿元，增长7.9%。开展184个行政村土地承包经营权确权登记颁证工作，确权登记面积超过100万亩，实现土地流转面积29.1万亩。公务用车改革顺利推进。不动产登记制度顺利实施。

（三）着力推进结构调整，经济结构逐步优化

战略规划先行。围绕“一极五业”发展战略，编制完成《中国（抚顺）经济产业转型振兴纲要》及《三年滚动计划》，将沈抚新区抚顺经济增长极、大生态、大石化、大材料、大能源、大旅游产业发展战略目标化、项目化。

工业做大做强。抚顺石化公司新增原油加工量30万吨、盈利水平在中国石油炼化企业中排名第三，缴纳税费留归地方20亿元、对地方财政贡献率达到25.9%。石化产业丰厚度提高,高新区有机化工、精细化工、合成新材料和橡塑蜡深加工4大产业集群项目达到120个、建成投产企业年产值近50亿元。抚顺特殊钢股份有限公司“三高一特”高附加值产品产量增长19%。《中国制造2025抚顺市实施方案》全面实施，开复工智能制造项目75个。航空配套、机器人配套和应急等新兴产业项目加快推进。

服务业提质增效。全市11个新建、在建亿元以上旅游项目全部列为省重点旅游项目，沈抚新城热高温泉养生世界被评为国家5A级养生度假温泉，抚顺县三块石森林公园被评为中国北方最美自驾旅游目的地，新宾县被国家农业部评为休闲农业和乡村旅游示范县，市旅游委被人社部和国家旅游局授予“全国旅游系统先进集体”称号。全年旅游总收入增长14%，接待游客人数增长13%。东北亚钢铁网平台实现交易额224亿元、增长13.6倍；中国琥珀网实现线上运营；顺城区被评为中国电子商务创新示范基地。举办各类会展和节庆活动18次，参展企业1241家，实现交易额27亿元。制定出台进一步化解房地产库存的实施意见，打通地质灾害区居民避险搬迁安置与去库存通道，成功举办春秋两季房交会，预计全年商品房销售面积、销售额分别增长36.6%、35.3%，增幅位居全省前列。

特色农业稳步发展。全年粮食产量60.79万吨。建成食用菌、中药材等百亩标准化园区27个、市级畜禽标准化养殖场20个。完成大伙房水源保护区有机农业示范基地有机食品认证3万余亩。新宾道地药材辽细辛被农业部认定为地理标志产品，大伙房有机鱼等获评2016年辽宁名牌农产品，旺清门镇、英额门镇和后安镇被授予2016年“辽宁特产之乡”称号。在全市12个村开展扶持村级集体经济试点，增强了农村集体经济实力。

（四）着力鼓励创新创业，发展新动能加快集聚

推动科技创新。在全省率先召开科技创新大会，7位“两院”院士和50位知名专家学者参会，与天津大学等签订战略合作协议6项，11个合作项目取得实质性进展，中国工程院张齐生院士工作站落户抚顺。全市新获批国家高新技术企业26家，引进域外创新平台3家，建成科技孵化器、“众创空间”等10家。抚顺市创新联盟为企业解决难题60余个，形成落地项目18个。大专院校、科研院所协调服务中心推进域内合作项目130余个，协调解决制约“三院三校”发展和创新问题50余件。

营造创新创业氛围。“市民业余学习行动”深受欢迎，创建市（区）级学习基地5个，授课128场次，参与市民1.04万人次，《中国教育报》进行了专题报道。开设“浑河讲堂”网络专栏，上传课程视频94节，网络点击量达18万人次。“浑河讲堂”被评为2016年全国特别受百姓喜爱的终身学习品牌。抚顺返乡大学生科技创业孵化基地被科技部命名为“星创天地”，

望花“双创”孵化中心和沈阳工学院大学生创业孵化基地被省科技厅命名为“云启众创”。新抚区、望花区荣获“省级创业型县区”称号。弘扬企业家精神和工匠精神，9位功勋企业家受到表彰。

（五）着力保障和改善民生，群众拥有更多获得感

重大民生工作取得突破。在时间紧、任务重、群众诉求多元、情况极其复杂的条件下，有力有序、如期顺利完成地质灾害区居民（6141户）避险搬迁安置工作，得到国家发改委、住建部等部委和省委、省政府充分肯定。成功破解榆林采沉区31方块安置历史遗留问题，榆林地区2894户居民喜迁新居。地质灾害区居民生命财产安全问题得到根本性解决。圆满完成保障性安居住房建设任务，棚改各项工作走在全省前列。

“三计划两工程”顺利实施。“青草计划”完成城区裸露地面绿化318万平方米。“照明计划”安装居民小区楼体灯具13454盏、小街巷路及广场照明设施569盏。“平坦计划”修补300余条小街小路、1万余处坑洼、15.8万平方米。“暖房子”工程完成改造面积307万平方米，工程量为过去4年总和的3.5倍，惠及5万多户居民。“温暖工程”新建改造供热管网170公里，覆盖700万平方米，惠及居民21万人。青草全覆盖、照亮回家路、脚下路平坦、温暖过冬天，这些看似小事、却与百姓生活息息相关。只要百姓受益、只要百姓认可，即使政府财力再困难，我们也一定要坚持下去，让更多的人民群众有更多的获得感。

社会保障水平不断提高。继续提高企业离退休人员养老金标准，千方百计确保全市41.4万企业离退休人员养老金按时足额发放。全面提升社会保障信息化水平和便民服务水平，“金保二期”工程上线运行。城乡低保标准提高5%和10%，农村五保对象供养标准平均提高7%。城镇登记失业率4.06%，零就业家庭保持动态清零。投放妇女创业小额担保贷款5亿元，惠及城乡9874户家庭。减少农村贫困人口1.5万人，销号60个贫困村。

社会事业协调发展。开工薄弱学校改造项目47个，205所义务教育阶段学校实行“阳光分班”。6所二级以上中医医疗机构设置“治未病”科，新农合政府补助标准由380元提高到420元。市博物馆（群众文化中心）改扩建工程启动。《地理标志产品——抚顺琥珀》省地方标准发布实施、结束了抚顺琥珀百年无标准的历史。成功承办三块石国际登山比赛、全国男排联赛辽宁队主场比赛等赛事，开展大型全民健身活动超过100项次，抚顺籍运动员韩天宇荣获“全国十佳运动员”称号。

社会治理水平持续提升。信访总量下降28%，刑事案件发案率下降8.4%，全年重大交通事故为零。开展“小凉快”区域性治理，查扣“小凉快”700余台次。完成易燃易爆危化品企业厂区装置、工艺管线、储运设施等重大危险源分布图绘制工作，提高了事故防范及救援的科学化和精细化水平，得到国务院安全生产巡查组和省安监局的充分肯定。安全生产形势总体稳定。食品药品安全监管体系逐步完善。

抗灾减灾能力切实增强。连续战胜3次强降雨袭击，转移安置群众超过5万人次，全市安全度汛。尤其是“7·25”强降雨，西露天矿北帮发生面积10万平方米、体积313万立方米滑坡，由于预报准确、应对得力，及时转移居民562户，无一人伤亡。

（六）注重城乡统筹协调，人居环境持续改善

生态环境进一步优化。拆除10吨及以下燃煤锅炉444台。淘汰黄标车和老旧车辆10618台。完成龚家沟地板城电网改造，消除了区域性重大火灾隐患；关停违规违法生产企业241家。关停榆林地区小塑料加工厂17家。城区空气质量优良天数增加24天。综合治理浑河支流河，大伙房水源保护区库区水质基本达到II类标准。完成造林面积12.87万亩，我市被授予“全国绿化模范城市”称号。

城市建设不断加强。新一轮城市总体规划修编（2013-2020年）完成省部联审。新抚区老工业基地搬迁改造进展顺利，完成章党街等海绵型城市基础设施建设。章党桥新建工程竣工通车，辽宁中环线（抚顺段）高速公路工程建设、国道202线（城区段）北移改造工程前期工作稳步推进。完成近10万户居民天然气置换、城区2万户老旧房屋室内供水设施改造。城市周边永久基本农田划定初步任务核实举证成果首批通过国土资源部审核。

宜居乡村建设积极推进。完成“一事一议”财政奖补村内道路389.6公里，新建改建农村公路72.3公里，治理河道66公里。建设畜禽粪便污水处理设施主体工程2个，完成农村改厕5798座。20个村、3个乡镇被评为省级生态村（镇），37个村成为全省美丽乡村示范村。

（七）加强政府自身建设，工作作风扎实改进

履职尽责，严格自律。坚持全面从严治党，强化对权力运行的制约和监督，扎实开展“两学一做”学习教育，认真整改中央巡视组“回头看”反馈问题，政府系统党的建设和党风廉政建设不断加强。

科学精细，求真务实。遵循规律，科学精细谋划抚顺转型振兴发展。顶住压力，坚决做实经济数据。敢于担当，公汽总公司债务风险、望花区帝景翰苑居民回迁等问题有效解决。不务虚功，想事、干事，坚持不懈干成事，机关作风更加务实、干事创业氛围更加浓厚。

遵纪守规，接受监督。定期向市委汇报工作，认真落实市委部署要求。实行政府常务会议审计、监察、法制部门全程参与制，确保决策依法合规。自觉接受市人大及其常委会的依法监督和市政协的民主监督，积极听取各民主党派、工商联、无党派人士和人民团体的意见建议。355件代表建议、361件政协提案全部办结。全面推行行政负责人出庭应诉制度，依法办理69件政府信息公开申请。及时回应网络舆情和群众诉求，全年办理群众诉求2.89万件。

质监、国家安全、海关、出入境检验检疫、烟草、保险、邮政、通信、电力、民族、宗教、司法、侨务、档案、供销、防震、老龄、残疾人等各项工作都取得了积极进展。市人防办被评为“全国人民防空先进集体”。我市获得全国“双拥模范城”称号。

各位代表：刚刚过去的2016年，我们坚决贯彻落实中央各项方针政策，抢抓机遇，变压力为动力、变劣势为优势、变机遇为成效，在困境中奋进、在拼搏中前行，谱写了城市转型振兴发展的新篇章。成绩来之不易，这是省委、省政府和市委正确领导，市人大、市政协大力支持和全市广大干部群众共同努力的结果。在此，我代表市人民政府，向全市各族人民，人大代表、政协委员，各民主党派、工商联、无党派人士、人民团体、离退休老同志，驻抚部队、武警官兵、公安干警、中省直单位和以抚顺石化公司为代表的为抚顺作出重大贡献的机关企事业单位，以及所有关心、支持抚顺发展的海内外朋友们，表示崇高的敬意和衷心的感谢！

看到成绩的同时，我们也要看到问题。去年我市地区生产总值、全社会固定资产投资等指标没有完成预期目标。既有营商环境不优和主动做实经济数据的原因；也有传统产业比重较大、新兴产业发展不充分、经济发展新动能不足等因素；还有部分公务人员思想保守、路径依赖、不愿作为、不敢担当、工作效率不高等问题；特别是占城市建成区42.5%的地质灾害影响区破坏严重、占全市国土面积57.46%的大伙房水源地保护任务艰巨、养老金缺口巨大、厂办大集体问题突出、政府债务负担过重、老龄化问题凸显等历史遗留问题和现实矛盾相互交织、并存叠加，别的城市没有的问题我们有，别的城市有的问题我们更加严重，这些极大的影响和制约了抚顺转型振兴。

对此，我们必须时刻保持清醒头脑，深刻认清抚顺面临的困难十分突出，深刻认清抚顺振兴发展的任务十分繁重，深刻认清抚顺要化解的矛盾和问题十分复杂，深刻认清抚顺在转型振兴的进程中正处在不进则退、不干则衰的关键时期。我们既要保持战略定力，又要只争朝夕，坚持问题导向，分类施策，以巨大的耐力和韧劲，科学谋划工作，扎实推进转型振兴。

二、2017年重点工作任务

2017年是党的十九大胜利召开之年，是推进供给侧结构性改革的深化之年，也是实施“十三五”规划、全面建成小康社会的关键之年。展望新的一年，我们对抚顺发展充满信心。

我们的信心来自于抚顺转型振兴面临的重大历史机遇。党中央、国务院相继出台一系列支持东北新一轮振兴的重大举措，必将有力促进抚顺转型振兴；省委、省政府将沈抚新区建设上升为重大区域发展战略，必将辐射带动抚顺发展，成为抚顺新的增长极。

我们的信心来自于对市情认识的不断加深。经过一年来的深度梳理，抚顺发展的优势劣势、存在的困难问题更加清晰，知耻后勇、扬长避短、攻坚克难将更有针对性。特别是通过积极向上反映抚顺老工业基地和资源枯竭型城市特殊性、典型性问题，国家和省支持抚顺化解矛盾的力度加大，影响和制约抚顺转型振兴的重大问题正在逐步破解。抚顺将踏上释重减负、轻装上阵的发展之路。

我们的信心来自于抚顺的发展思路和工作重心更加明晰。市委确定了“一极五业”发展战略，坚持以项目调结构、促发展，形成了策划包装一批、洽谈推进一批、签约落地一批、开工建设一批、竣工投产一批的项目建设良性循环，投资超过百亿元的清原抽水蓄能电站项目全面开工，投资超过200亿元的沈佳高铁和沈吉铁路(城区段)外迁工程年内有望全面启动，抚顺转型振兴的方向明确、支撑有力。

我们的信心来自于风清气正的政治生态正在逐步形成。通过落实中央巡视反馈意见、严肃查处拉票贿选案等，全市政治生态得到净化，弘扬正风正气、激励干事创业的导向更加鲜明，集聚正能量、释放精气神、全力促振兴的氛围已经形成。

我们的信心来自于全市人民对美好未来的热切期盼。这种期盼凝聚了230万人奋进的力量、前行的勇气。只要我们坚定信心、团结一心、不忘初心，把面临的重大机遇和有利条件充分利用起来，把经济发展的巨大潜力充分挖掘释放出来，把全市广大干部群众热爱家乡、共创美好未来的积极性调动起来，抚顺转型振兴的目标就一定能够实现！

今年政府工作的总体要求是：以党的十八大和十八届三中、四中、五中、六中全会精神为指导，紧紧抓住新一轮东北振兴重大机遇，认真贯彻中央、省经济工作会议和省、市第十二次党代会精神，坚决落实全面从严治党要求，继续坚持稳中求进工作总基调，以新发展理念和“四个着力”为引领，以转型振兴为主线，以改革创新为动力，以“项目年”为抓手，深入推进供给侧结构性改革，大力实施“一极五业”发展战略，壮大实体经济，不断提升经济发展活力和内生动力，形成多点支撑、多元发展、多业并举的良好发展态势，努力开创抚顺老工业基地振兴新局面，为全面建成小康社会奠定坚实基础。

全市经济社会发展的预期目标是：地区生产总值增长6.5%，全社会固定资产投资增长12%，规模以上工业增加值增长7%，社会消费品零售总额增长9%，一般公共预算收入增长5.5%，城乡居民人均可支配收入增长6%，城镇登记失业率控制在5%以内。

上述预期目标的确定，主要是按照省委、省政府的明确要求，与“十三五”规划和全面建成小康社会目标相衔接、与提高人民生活水平相适应、与全国全省发展保持同步。

今年要重点抓好以下工作。

（一）继续完善体制机制，积极构建科学精细的经济社会管理模式

深化“放管服”改革。全面对标国内先进地区，继续取消和下放一批市级行政职权事项，减少和规范涉企收费及审批评估事项，全面落实“双告知”和“双随机一公开”制度，逐步理顺政府与市场的关系。全面推行“一个窗口受理、一站式办理、一条龙服务”，重点探索“受审分离、审管分离”运行机制。建立市、县（区）、乡镇（街道）、村（社区）四级服务网络体系，整合分散在各部门各行业的80多条便民服务热线为“12345”一键通市民诉求和服务平台，提升服务水平，更加惠民利企。

打造良好营商环境。深入推进“法治抚顺”、“信用抚顺”、“效率抚顺”建设，以政府诚信、高效带动社会诚信、高效。加强对各种所有制组织和自然人财产权的保护，构建“亲”、“清”新型政商关系，探索实行企业投资项目承诺制，降低税费和要素成本，增强企业在抚发展信心。严格规范行政执法行为，重点解决重复多头执法、滥用自由裁量权、违法进行行政强制等执法扰民扰企问题。对损害营商环境的行为，严厉查处、严肃追责。

深化国资国企改革。继续抓好国有资产集团组建和运营工作，以管资本为主构建国资监管新体制，全面提高国资运营质量和效益。支持在抚央企发展混合所有制经济。全面摸清政府行政资产底数，强化对资产配置、使用、处置、收益等环节监管，整合优化政府行政资源取得突破性进展。

加大政府机构和事业单位改革力度。积极跟进国家和省政府机构改革进程，全面优化政府职能配置。加快检验检测认证、机关后勤服务、培训疗养等机构及承担行政职能事业单位改革，推进事业单位机构整合和编制大幅精简。完成事业单位用车制度改革。

加快财税体制改革。进一步落实营改增、资源税改革等政策，全面实施财政零基预算改革，严格控制债务总量和新增债务规模，通过组建国有资产集团、发行置换政府债券、与社会资本合作（PPP）等方式，有效缓解政府当期债务压力，高度关注防范和化解政府债务风险。

全面扩大对外开放。积极拓展“一带一路”沿线市场，深化与中国对外承包工程商会和军贸企业等合作，推动我市产品、装备、技术、标准和服务“走出去”。加快对外贸易模式转型创新，大力发展外贸服务综合体和跨境电商平台。

（二）继续推进结构调整，以项目建设促转型振兴

紧紧围绕“项目年”建设，持之以恒抓项目，努力形成全方位策划项目、招商引资、争取支持，全领域开拓新思路、实施新举措、推进新项目，全社会谋发展、促转型、促振兴的生动局面，走出一条多点支撑、多元发展、多业并举、协调可持续的转型振兴发展之路。

加大项目储备和招商力度。重点围绕国家支持东北振兴系列政策措施、创建国家可持续发展议程创新示范区、创建国家首批老工业城市和资源型城市产业转型升级示范区，围绕实施“一极五业”发展战略，继续策划包装更多向上争取、对外招商和与社会资本合作的重点项目。坚持领导带头，按照县区招商主体、市直部门服务主体和统筹推进重大项目的工作格局，重点面向长三角、珠三角、北京等地，聚焦“一极五业”开展主题概念招商，围绕大石化、大材料等原料资源实施产业链招商，依托整合盘活闲置土地、厂房等进行要素招商，着眼文化、体育、旅游、休闲等领域实施多元招商。全年域外引资到位额150亿元，实际利用外资实现新突破。

做强存量调整结构。强力推进大石化产业发展，加快与抚顺石化公司共建产业园区，拉长拓宽产业链条，做大做强石化新城化工及精细化工等下游产业；鼓励支持抚顺石化公司深挖潜力，加快实施投资83亿元的技术改造项目、盘活原值39亿元的低效闲置资产。全力支持抚顺矿业集团转型发展。强力推进大旅游产业发展，围绕建设中国旅游名城目标，保护、利用工业遗产资源，加快推进“东北记忆”工业文明主题广场建设，着力发展工业旅游，创建抚顺独一无二的旅游品牌，打造“遥远的赫图阿拉”满族文化旅游目的地，按规划联创国家5A级景区，争创国家全域旅游示范市。

做大增量优化结构。全力向上争取沈佳高铁和沈吉铁路（城区段）外迁工程以及国道202线（城区段）北移工程年内开工建设，结束抚顺没有高铁和沈吉铁路纵贯城区的历史，优化城市空间布局，释放更多发展空间。强力推进大能源产业发展，全力推进清原抽水蓄能电站项目建设，同步促进与此相关的旅游和电力配套产业发展，创造更多的就业机会，带动开发式扶贫；规划建设总投资超百亿元的光伏领跑者基地项目，加快推进垃圾焚烧发电项目，促进煤电等传统能源转型升级。加强与国药集团等大型央企合作，推动新宾县大化国瑞股权合作、沈抚新城医疗健康产业园等项目开工建设。积极引进生活消费品生产项目，培育和发展消费品工业。全年开工建设亿元以上重点项目155个、当年完成投资137.6亿元。

紧跟新兴产业发展步伐。加强与大型民营企业合作，全面启动顺城区城市创新创业综合体项目，加快10个特色小镇规划。强力推进大生态产业发展，坚持保护优先、适度合理开发，重点实施生态恢复整治、自然资源保护、人居环境优化、美丽乡村建设、绿色城镇化建设工程。强力推进大材料产业发展，积极发展化工新材料、冶金新材料和新型建筑材料等新兴产业。加快推动东北亚钢铁网、东北亚木业交易中心等电子商务交易平台及望花区百科物流园建设。

深入推进农业供给侧结构性改革。实施新一轮农村电网改造工程，新增节水灌溉面积1万亩、高标准基本农田8.54万亩。稳步推进农村集体产权制度改革，计划实施土地承包经营权确权登记颁证面积88万亩。推进农业种植结构调整，提高供给质量，玉米种植面积调减2.1万亩，设施蔬菜产量10.57万吨，继续扩大设施农业、高效特色农业和绿色有机农业种植规模。新建生态型特色农产品园区20个、市级畜禽标准化养殖场20个。深入实施“互联网+营销”战略，积极培育和引进一批农产品深加工龙头企业，建设农产品交易中心和面向沈阳市场的特色农产品配送中心。规范农业综合开发项目招投标方式，确保资金充分利用。村级集体经济发展试点扩大到21个村，推动农业融合发展，带动农民增收致富。

加快发展金融业。坚持金融服务实体经济的本质，全面推进产业与金融结合。积极搭建银企对接平台，完善融资担保体系，促进金融产品创新，充分利用多层次资本市场，进一步拓宽企业融资渠道。加快沈抚新城产业金融集聚区建设，提升新华金融商贸集聚区服务功能，在新抚区打造金融创新产业基地，促进各类金融要素集聚。积极筹建抚顺农商行和新宾农商行。设立支持石化等重点产业发展引导基金和产业（创业）投资基金。持续完善地方金融监管体系，防范金融风险。全年金融业增加值增长8%。

积极消化房地产库存。继续畅通棚户区改造、新型城镇化与房地产去库存的通道；创新房交会形式，放宽住房公积金使用条件，活跃房地产交易市场，加快建立符合抚顺市情、适应市场规律的房地产平稳健康发展长效机制。全年商品房销售面积增长12%。

（三）继续鼓励创新创业，加快培育经济发展新动能

完善科技创新体系。依据《中国落实2030年可持续发展议程创新示范区建设方案》，积极创建国家可持续发展议程创新示范区。深入实施科技型中小企业成长、高新技术产业倍增计划，重点培育扶持一批创新型领军企业。以抚顺高新技术产业园区为依托，整合科研院所、大专院校等各类科研资源，启动国家级高新技术产业开发区争创工作。充分发挥科技协调服务中心作用，推进企业与科研院所和大专院校合作，加快企业研发中心、校企联盟、产业技术创新联盟建设。积极引进风险投资等各类创新投资基金，加快形成“政产学研金用”相结合、全社会共同参与的科技创新体系。开展重大产学研合作10项，实施重大技术创新20项。

加强众创平台建设。加快发展总部基地创投中心、东北振兴网络投融资平台、返乡大学生科技创业孵化基地、乐创空间等众创平台，举办首届抚顺创新创业大赛、第二届“为抚顺而来”国际大学生设计大赛，形成各类创新主体互促、科技精英与“民间草根”并肩的生动局面。积极向内挖潜，全力扶持培育本地企业研发新项目。落实人才计划，吸引留住人才，鼓励小改小革、点滴创新。继续推进“市民业余学习行动”，在全社会营造浓厚的学习、向上氛围，为大众创业、万众创新打下坚实基础。

加大创业扶持力度。建立和完善创业孵化基地7.9万平方米、为320户企业提供创业场地。对未进入创业孵化基地、首次自主创业并带动就业的高校毕业生、退役士兵及就业困难群体，给予创业场地补贴。

（四）继续保障和改善民生，努力创造宜居乐业的经济社会发展环境

提高社会保障救助能力。全力确保企业离退休人员养老金按时足额发放。适时提高城乡低保救助和农村五保供养标准。鼓励城镇职工、城镇居民、困难企业退休人员参保，努力实现医疗保险全覆盖。深入推进医疗救助“一站式”结算服务，继续开展“精神康宁”等慈善救助项目。多渠道稳定和扩大就业，城镇新增就业2.1万人。坚决打赢扶贫攻坚战，新完成1.33万建档立卡贫困人口稳定脱贫，实现58个贫困村和2个省扶贫开发重点县有序退出。

改善人居条件。新建改建燃气管网25公里。实施城市积水点改造，提升城市抗灾能力。新增棚户区改造2627户，实施“暖房子”工程50万平方米，改造老旧房屋室内供水设施1万户。大力推进城区小街小巷道路和村内道路改造。扩大“青草计划”、“照明计划”、“平坦计划”实施范围。

大力发展社会事业。加快发展普惠性学前教育，推动义务教育资源均衡配置，深化普通高中课程改革，增强职业教育服务地区发展的能力。启动城市公立医院综合改革，推进医保、医疗、医药“三医”联动，构建分级诊疗就医新秩序，启动“卫生城市、健康城市”双城联创。积极应对人口老龄化，加快构建多层次养老服务体系。深入挖掘抚顺文化内涵，大力弘扬雷锋精神，加快发展文化事业和产业，重塑抚顺文化自信。深入推进公共文化设施均等化。恢复建设界藩城、萨尔浒城等清前王城遗址，启动建设元帅憾林石刻展区。积极发展琥珀产业。办好大型体育赛事。推动大型公共体育场馆面向社会免费、低收费开放。做好争办第15届（2024年）全国冬季运动会的前期工作。

保护生态环境。牢固树立绿水青山就是金山银山的理念，着力推进污染防治。全面推行河长制，深入实施大伙房水源保护区综合治理，整治城市建成区黑臭水体，严厉打击超标排放污水等违法违规行为。大力实施气化抚顺、绿色交通、清洁能源推广、秸秆综合利用和工业提标改造等工程，淘汰10吨及以下燃煤锅炉222台，淘汰黄标车及老旧车辆4486辆，新增集中供热并网面积393万平方米，加大对重点污染企业和区域的环保整治力度，全年空气质量达标天数275天。严肃查处乱砍滥伐林木、破坏和非法使用林地等违法行为，全年人工造林10.15万亩，封山育林0.91万亩。

坚持规划引领。遵循城市发展规律，从地下、地上、空间三个层面，将以人为本、传承历史、尊重自然等理念融入城市规划。结合沈佳高铁建设和沈吉铁路（城区段）外迁、国道202线（城区段）北移等，尽快启动相关区域空间发展规划研究。启动甲邦通道规划建设。启动对临江路东延、新城路东延、中央大街人行景观桥和葛布老桥改造等全市路桥网络的论证和规划研究。

完善城市功能。抚顺资源枯竭型城市可持续发展示范市规划展示馆、国家地理国情监测中心投入使用。建成市博物馆、市档案馆和城市指挥中心。加快东林儿童运动公园和户外体育运动中心等项目建设，全面改造城建档案馆和劳动公园动物园。对中央下放移交的国有关闭破产企业“三供一业”进行全面维修改造。启动安城通道、公共停车场规划建设，进一步提升城市形象、保障城市发展需要。

提升管理水平。全面启动城市建成区违法建设专项治理工作五年行动。加快拆除地质灾害区避险搬迁安置居民腾空房屋。加强智慧城市建设，加快数字化城管启用。规范调整路域导览导视系统和城市交通指示牌。继续依法依规开展“小凉快”专项治理。全面加强物业管理。大力实施城市环境卫生清洁治理和居民小区环境综合整治。深入开展农村环境综合整治，推广农村垃圾分类减量处理办法，改造农村卫生厕所3720座。

强化社会治理。推进城市社区网格化管理，构建立体化社会治安防控体系升级版，建立矛盾纠纷多元化解机制。开展信访稳定百日攻坚战。做好法律援助工作。完善并严格落实安全生产责任制，严防重特大事故发生。加强食品药品安全监管，创建食品安全城市和食品安全放心县（区）。以城区为单位，继续推进文明殡葬改革。

民生是发展的目的，人心是最大的政治。一心一意办实事、科学精细惠民生，没有最好只有更好。尽管当前财政收支矛盾日益突出，但是政府关注民生的态度坚定不移、改善民生的力度持续久远、保障民生的投入只增不减，将竭尽所能，让全市人民生活更美好！

（五）全面加强政府自身建设，努力开创政府工作新局面

把牢政治方向。全面贯彻落实党的十八届六中全会精神，坚决维护以习近平同志为核心的党中央权威和集中统一领导，牢固树立政治意识、大局意识、核心意识、看齐意识，始终在思想上政治上行动上与以习近平同志为核心的党中央保持高度一致。

增强前列意识。站位全国、全省，对标先进地区，转变思想观念，创新工作举措，勇于超越，追求卓越，努力在关键领域、重点工作上走在全省、全国前列。

强化责任担当。继续坚持科学精细的思想方法和工作方法，干当前、想长远，面对难题敢于攻坚，谋划工作尊重规律，盯紧目标持续发力，干一件、成一件，以干事创业凝聚人心，以干成事业提振信心。积极推行容错免责机制、完善绩效考评体系，为干事者撑腰、对担当者负责。

主动接受监督。坚持用权为公、执政为民，自觉接受市人大及其常委会依法监督、市政协民主监督，倾听社情民意，回应社会关切，改进政府工作。

坚持廉洁自律。坚决落实全面从严治党要求，严守党风廉政建设各项规定，加强对重点行业和领域、关键岗位和环节的审计监察，严肃查处违规违纪行为，以党风政风转变带动社会风气转变。

各位代表、各位委员，同志们，加快抚顺转型振兴是全市人民的共同心愿，也是时代赋予我们的重大责任和光荣使命。让我们在省委、省政府和市委的正确领导下，既保持战略定力，又只争朝夕，以敬业担当的精神诠释对党的忠诚，以科学精细的作风积跬步而至千里，以躬身实干的行动促进转型振兴，用抚顺人民的共同事业凝聚人心，汇集230万抚顺人民的智慧和力量，铸就抚顺新的辉煌！
